# OCDD: an obesity and co-morbid disease database

**DOI:** 10.1186/s13040-017-0153-5

**Published:** 2017-11-21

**Authors:** Indrani Ray, Anindya Bhattacharya, Rajat K. De

**Affiliations:** 1 Machine Intelligence Unit, Indian Statistical Institute, 203 B.T. Road, Kolkata, 700108 India; 2 Department of Computer Science and Engineering, University of California, 9500 Gilman Drive, La Jolla, San Diego, 92093 CA USA

**Keywords:** Functional enrichment, NAFLD, Type 2 diabetes, Endometrial cancer

## Abstract

**Background:**

Obesity is a medical condition that is known for increased body mass index (BMI). It is also associated with chronic low level inflammation. Obesity disrupts the immune-metabolic homeostasis by changing the secretion of adipocytes. This affects the end-organs, and gives rise to several diseases including type 2 diabetes, asthma, non-alcoholic fatty liver diseases and cancers. These diseases are known as co-morbid diseases. Several studies have explored the underlying molecular mechanisms of developing obesity associated comorbid diseases. To understand the development and progression of diseases associated with obesity, we need a detailed scenario of gene interactions and the distribution of the responsible genes in human system.

**Results:**

Obesity and Co-morbid Disease Database (OCDD) is designed for relating obesity and its co-morbid diseases using literature mining, and computational and systems biology approaches. OCDD is aimed to investigate the genes associated with comorbidity. Several existing databases have been used to extract molecular interactions and functional annotations of each gene. The degree of co-morbid associations has been measured and made available to the users. The database is available at http://www.isical.ac.in/~systemsbiology/OCDD/home.php

**Conclusions:**

The main objective of the database is to derive the relations among the genes that are involved in both obesity and its co-morbid diseases. Functional annotation of common genes, gene interaction networks and key driver analyses have made the database a valuable and comprehensive resource for investigating the causal links between obesity and co-morbid diseases.

**Electronic supplementary material:**

The online version of this article (doi:10.1186/s13040-017-0153-5) contains supplementary material, which is available to authorized users.

## Introduction

Obesity is the new epidemic of 21st century [1]. Being a platform of numerous other diseases including type 2 diabetes, asthma, cardio-vascular disease and several cancers [2–5], obesity is considered as one of the most prevalent forms of conditions for the most life threatening diseases. According to the World Health Organization (2014), there are around 2 billion adults worldwide who are overweight, out of them 670 millions are nearly obese [body mass index (BMI) > 30 kg/m^2^], and 98 millions are severely affected by obesity [body mass index (BMI) > 35 kg/m^2^]. Obesity reduces life expectancy.

The number of co-morbid diseases that are associated with obesity is large and many of them are already reported in different research articles [6, 7]. According to the medical history of the patients, obesity is known to increase the risk of developing type 2 diabetes by 60%, hypertension and coronary-heart diseases by 20%, and cancers by 30% [8]. Other co-morbidities include gall-bladder disease, fatty liver and osteoarthritis [9]. Obesity is commonly known as a lifestyle driven disease. However, there exists significant evidence in literature regarding the possibility of altered molecular interactions mediated by obesity [10]. Earlier, gene network based *in silico* analyses have revealed the molecular links among obesity, hypertension and cardiovascular diseases, and type 2 diabetes [11]. Recent systems biology approaches have been exploring the detailed molecular interactions in obesity based on gene expression, novel marker genes, structural variants, copy numbers, polymorphisms and protein-protein interactions (PPIs) [10, 12]. Identifying the network hubs from gene and PPI networks is a very common step in the recent study for understanding the diseases and co-morbidity [13, 14]. For example, the analyses of gene network for adipose tissue and inflammatory diseases have revealed a significant overlap between the networks and their hub genes (centers) [10, 13, 14].

Thus systematic accumulation and organization of data related to association among obesity and co-morbid diseases, is necessary in the form of a database. The fast growing number of the research articles that are reporting the molecular links among obesity and co-morbid diseases, is raising the need for systematically accumulating and organizing the co-morbidity study and developing databases. The existing databases that are known to store the co-morbidity links among obesity and other diseases are mainly focusing on single disease, e.g., T1Dbase database [15] for type 1 diabetes and T2D-Db [16] for type 2 diabetes. Text-mined Hypertension, Obesity and Diabetes candidate gene database (T-HOD) is another example of disease specific co-morbid database.

T-HOD is a text mining database, containing 837, 835 and 821 candidate genes mediating hypertension, obesity and diabetes, respectively. The genes are ranked based on the number of their appearances, protein-protein interactions and single-nucleotide polymorphisms (SNPs). T2D-Db (Type 2 diabetes Database) is a comprehensive web resource that provides information of almost all known molecular components involved in T2D in human, mouse and rat. Information on candidate genes, SNPs (Single Nucleotide Polymorphism) in candidate genes or candidate regions, genome wide association studies (GWAS), tissue specific gene expression patterns, EST (Expressed Sequence Tag) data, expression information from microarray data, pathways, protein-protein interactions and disease associated risk factors or complications have been structured in T2D-Db. T1DBase contains molecular genetics and biology of type 1 diabetes (T1D) susceptibility and pathogenesis. It has a unique attribute called *β* cell genome atlas.

However, the need for a database for storing the information related to molecular links among obesity and all the co-morbid diseases is yet to be fulfilled by the existing databases. Here, we have designed Obesity and Co-morbid Diseases Database (OCDD) as a resource. The data for OCDD have been curated by applying a computational pipeline that performs both text mining on literature databases, such as Pubmed [17], GENECARD [18], and biological network mining on systems biology databases, such as STRING [19, 20]. OCDD is based on a pipeline that has been followed by T2D-Db and iCOD [16, 21]. The co-morbidity of diseases is explained as co-existance of two or more diseases whenever they affect the same individual [22]. Co-morbity is more significantly related to phenotype data while the real cause of co-morbidity can only be found by exploring the molecular interactions beneath the concerned diseases. Patient data are very useful in terms of establishing the co-morbidity relationship among obesity and other diseases. Hidalgo et al. have developed a dynamic network on disease pheotype (PDN) using 30 million patient data [23]. Using this network, we have validated our obesity related co-morbid disease list. Gene ontology data from DAVID [19] and GOrilla [24] have been used for annotating the functionality of groups of common genes that are associated with obesity and co-morbid diseases. The most attracting feature of OCDD is that apart from being a database of curated data from literature, and other primary and secondary data resources, it stores the results of an analysis of our systems biology based computational pipeline, which provides a clear insight of the role played by the genes that are most likely the major players in obesity and co-morbid diseases.

## Database features

OCDD database has been designed for showcasing the molecular interactions on the onset of the co-morbidity of obesity and 26 diseases. Following are the key features of OCDD.

- A comprehensive list of diseases is provided, which are repeatedly reported as co-morbid to obesity. A text mining on literature databases has been made for identifying the co-morbidity to obesity.
- Integration of literature mining results in the disease pathway data from KEGG (Kyoto Encyclopedia of Genes and Genomes) database.
- The co-morbidity to obesity has been measured based on statistical significance test over the search results that have been obtained from text mining and systems biology databases. A higher number of search results that are common between a disease and obesity, has been assigned a higher score for indicating a stronger co-morbidity with obesity.
- The list of obesity associated co-morbid diseases is further validated using *ϕ* and RR scores available in existing Phenotypic Disease Network (PDN) [23].
- The common genes that are known for their role in obesity and a co-morbid disease, are listed and annotated. All the biological processes, molecular functions and cellular components that are found enriched in the common gene groups, are listed in the database tables.
- All the gene-gene interaction networks have been formed and listed for the list of common genes associated with obesity and each of the co-morbid diseases.
- The network hub genes have been identified from the interconnections.

## Method

Here we describe the method that we have adopted while designing OCDD. The patient data in OCDD has been taken from the published results of experiments conducted in Barabasi’s lab [23].

### Data collection

**Fig. 1:**
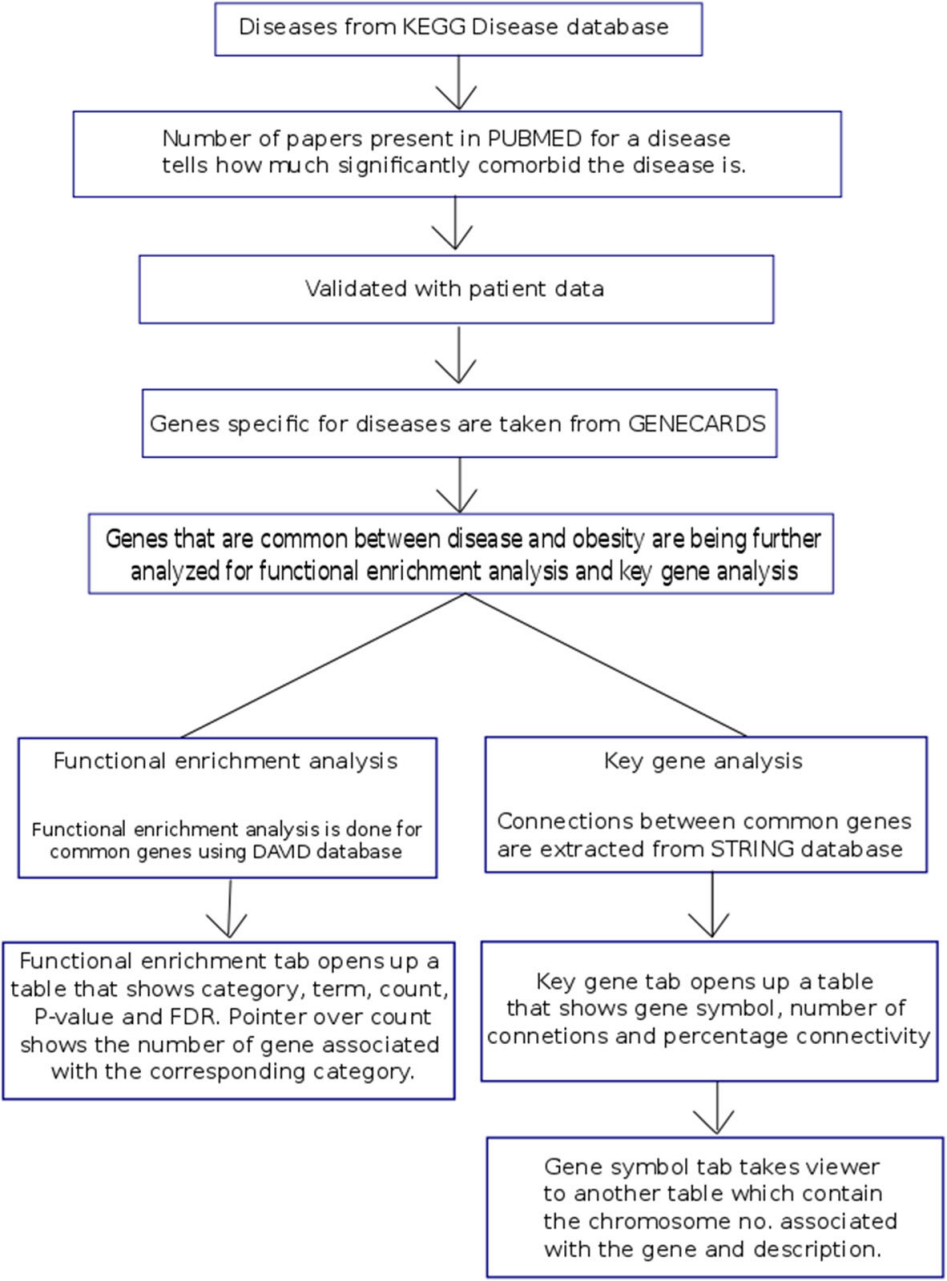
Flowchart

In this present work, we have collected data by mining different databases. The flowchart (Fig. 1) depicts how and where the data have been collected from. Then the data have been analysed for finding meaningful biological connections to understand the molecular mechanism lying behind obesity and co-morbid diseases.

### The obesity associated co-morbid diseases

KEGG (Kyoto Encyclopedia of Genes and Genomes) [25] is a comprehensive data resource for disease related biological pathways and molecular functions. We have collected the names of all the diseases from KEGG database. Then we have performed text mining on PubMed [17] by using the term “obesity” and each disease name as keywords. For keyword search on PubMed, we have used Biopython [26, 27] data search utility. From nearly 22 million current entries of biomedical articles in PubMed, all the abstracts that have been matched with the disease name as a key word, have been retrieved. For computing the statistical significance of each key word search, we have performed Fisher exact test on the observed number of key word matches. The *P*-values from Fisher exact test have then been adjusted by Bonferroni correction for multiple test adjustment. All the diseases with the adjusted *P*-value < 0.05 have been identified as co-morbid to obesity in OCDD database. The procedure of calculating Fisher exact test is provided in supplementary material.

### Validation with patient data

Co-morbidity refers to the co-occurrence of two diseases in a significant number of patients. Co-morbidity does not elucidate the molecular connections between co-occurring diseases. However, the modern science drives exploring the attributes that cause the co-occurrences of diseases. For this purpose, it is mandatory to validate the above obesity associated co-morbid disease list with patient data. Hidalgo et al. have developed a phenotypic disease network (PDN) on ICD-9 diseases using 30 million patient data [23]. Here, each disease in ICD-9 disease list has been analyzed for its comorbidity with other diseases, and two parameters, called *ϕ* value and RR score, have been calculated for understanding the degree of co-morbidity.

Consider a pool of *N* patients diagnosed with disease A and/or disease B. Let *I*_*A*_, *I*_*B*_ and *C*_*AB*_ be the numbers of patients diagnosed with disease A but not B, disease B but not A, and with both diseases A and B, respectively. Then *C*_*AB*_/*N* represents the chance of diagnosing both the diseases A and B in a single patient. On the other hand, *I*_*A*_/*N* and *I*_*B*_/*N* be the chances of diagnosing disease A but not B and disease B but not A in a single patient. Now, Relative Risk (*RR*_*AB*_) score for diseases A and B is defined as [23]

1 $$ \begin{aligned} RR_{AB} &= \frac{(C_{AB}/N)}{(I_{A}/N) \times (I_{B}/N)} \\ &= \frac{N \times C_{AB}}{I_{A} \times I_{B}} \end{aligned}  $$

and *ϕ*-correlation as [23]

2 $$ \phi = (RR_{AB}-1)\sqrt{\frac{I_{A} \times I_{B}}{(N-I_{A}) \times (N-I_{B})}}.  $$

The term *RR*_*AB*_>1 indicates that the chance of diagnosing both diseases A and B in a single patient is more than the joint probability (chance) of occurring disease A but not B and disease B but not A, in a single patient. In other word, *RR*_*AB*_>1 and thereby *ϕ*>0 signify that diseases A and B occur more frequently than they do by chance.

### Functional enrichment analysis

For each co-morbid disease, we have searched GENECARDS [18] database to retrieve the list of genes that are known to cause a disease as reported in biomedical literature. Sets of disease causing genes that are common with obesity genes have then been stored in OCDD as co-morbidity gene lists. Each co-morbidity gene list has then been put into the search engine of DAVID [19] for finding the functional annotation of the genes. All the biological processes, molecular functions and cellular components that are found enriched with adjusted *p*-values < 0.05, have been listed under function table tab in the database.

### Key gene analysis

A network of genes mediating obesity and a co-morbid disease is constructed using the resources and information from STRING database. STRING database provides all known and predicted associations among proteins/genes. These associations are scored and integrated, resulting in comprehensive protein networks covering more than 1100 organisms [20]. It considers complete knowledge of all direct and indirect interactions, i.e., neighborhood, co-expression, co-occurrence, gene-fusion, homology, database incorporation, textmining and experiments [20]. Finally, the key genes or hub genes have been extracted from the aforesaid networks. Here, we consider connections strictly associated with *Homo sapiens*, with scores greater than 0.9, to construct the above network. In order to find the key gene of a network, the genes for each disease are sorted based on the number of connections. The highly connected genes of a disease-obesity network is considered to be the key genes. Percentage connectivity is calculated on the basis of the number of connections. For each gene, chromosome number and description of the gene are provided in the database.

## Results

The functional enrichment and gene interaction networks have further been analyzed for elucidating the molecular mechanisms. The top 5% connected genes of each disease have been considered for further analysis to find the enriched GO terms.

### Distribution of top 5% connected genes

**Fig. 2:**
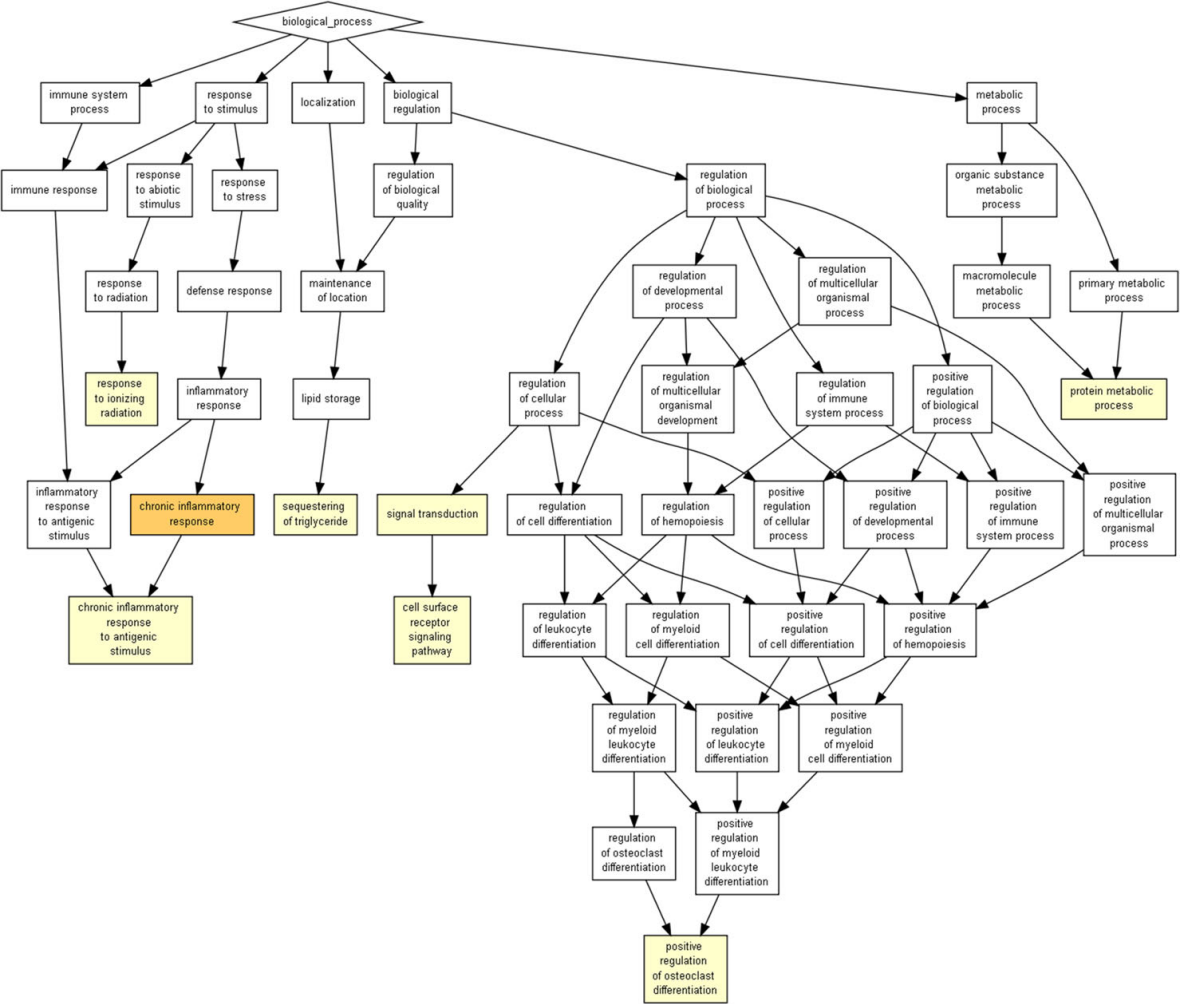
Distribution of 225 genes based on GO terms

The top most connected genes of each disease, i.e., the list of 225 top 5% connected genes from 26 co-morbid diseases are further analyzed on the basis of gene ontology terms (GO terms). The prominent genes are INS, LEP, VEGFA, TP53, STAT3, IL6, NFKB1. These 225 genes are clustered based on GO terms to find out the biological processes that are enriched with GO terms (Fig. 2). GOrilla is a web-based GO analysis tool with unique features to identify enriched GO terms in a list of genes. In GOrilla, the user does not require to provide any explicit target and background sets. GOrilla employs a flexible threshold in statistical approach to discover GO terms that are significantly enriched with a low *p-value* [24]. The list of 225 top 5% connected genes are analyzed by GOrilla. The output forms a tree of biological proesses with colour coding to understand respective biological processes and the extent of the biological processes being enriched with the genes. The result shows that the genes are mainly associated with regulation of protein kinase activity (GO:0045859), chronic inflammation response (GO:0002544,GO:0002439), sequestering of trigycerides (GO:0030730), protein metabolism (GO:0019538), positive regulation of osteoclast differentiation (GO:0045672), and response to ionizing radiation (GO:0010212). The most highly enriched process is chronic inflammatory response.

Obesity is always associated with chronic low level inflammation. The molecules from protein kinases are the agents of cross-talks among different systems present in our body. It is evident that the change in their regulation affects the end organs severely. Obesity disrupts the immunometabolic homeostasis of the system and the signaling molecules to act as the cross talkers. Sequestering of trigycerides is related to fat deposition and osteoclast differentiation is highly affected by obesity [28]. Even it is assumed that osteoporesis is nothing but obesity of bones [29]. Metabolism of proteins is also altered with the onset of obesity and hyperinsulinemia [30]. We have got no clue about how response to ionizing radiation is related to obesity.

## Discussions

The gene networks of type 2 diabetes, non-alcoholic fatty liver diseases and endometrial cancer show how the genes are densely connected. These three networks have been analyzed in the following sections.

### Type 2 diabetes

Type 2 diabetes is one of the prominent co-morbid diseases to obesity. A total of 561 genes have been found to be common between obesity and type 2 diabetes. Functional enrichment analysis of these 561 gene have been done. Out of 561 genes, we have found connections among 496 genes to draw the gene interaction network. The top most connected genes correspond to cell surface receptor proteins, signalling molecules, protein kinase molecules, and molecules from the immune system and hormone adiponectin. Several metabolites of fat metabolism, including acyl coA, ceramides, diacylgycerols along with FAs, act as signalling molecules. These signaling molecules activate a large number of kinases. These kinases phosphorylate serine of insulin receptor substrate (IRS) to stop insulin signalling.

Genome loci based data and population study support that highly connected genes, like ADIPOQ, MAPK3, PLCG1 and APPL1 in the database, play an important role in insulin resistance development [31, 32]. ADIPOQ or adiponcetin is an important hormone from adipocyte, whose reduction in secretion causes insulin resistance. Adiponectin signaling induces insulin sensitivity [33]. APPL1 mediates adiponectin signalling through Yin-Yang regulation [34].

Many genes involved in the immune system are present in this top connected gene list, which includes IL-6, TFN- *α* and IFN- *γ*. Previous investigations show clear connections in obesity, inflammation and type 2 diabetes [35]. Functional enrichment analysis also unveils that obesity with type 2 diabetes leads to many other diseases like colorectal cancer, bladder cancer, endometrial cancer and Alzheimer’s disease [36–38].

### Non alcoholic fatty liver diseases

Non alcoholic fatty liver diseases (NAFLD) form other prominent co-morbid diseases to obesity. NAFLD is a series of diseases starting from simple steatosis to steatohepatitis, followed by advanced fibrosis, and finally cirrhosis due to fat deposition in hepatocytes and subsequent increase in liver weight [39]. Simple steatosis is characterized by excessive fat accumulation, causing inflammation, cell death and scarring. Intermediate stages are defined by focal hepatic inflammation in acinar zone 3. In cirrhosis, there is presence of scar tissue and regenerative lumps that have been generated after several efforts to repair damaged tissue [39]. According to the database findings, a total of 176 genes have been found common between obesity and non-alcoholic fatty liver diseases. Out of these 176 genes, 137 genes have been found in the gene interaction network. The top most connected genes include INS, LEP, IL6, STAT3, PPARA and PPARG. Many scientific articles clearly state the link between insulin, leptin and NAFLD [40–42].

### Endometrial cancer

Endometrial cancer is the uncontrolled growth of inner lining of uterus, i.e., endometrim. Studies have shown that in more than 40% cases, overweight is related to endometrial cancer. Alterations in SHBG (sex hormone-binding globulin), progesterone and estrogen due to excess weight induce endometrial cancer [43]. A total of 482 genes has been found to be common between obesity and endometrial cancer. A densely connected gene interaction network has been built with 416 genes including highly connected genes, like insulin receptor gene (IRS), insulin growth factor gene (IGFBP5), intracellular adhesion molecule gene (ICAM), and many genes involved in the immune system (IL, NFKB, TLR, CASP). This proves that secretion of proinflammatory adipokines and adhesion molecules increase with the onset of obesity. These molecules play a vital role in cancer development.

Excess weight is linked with insulin resistance, which is developed as a result of increased free fatty acids in plasma. Insulin resistance affects IGF-I/IGFBP system [44, 45]. Obesity and insulin resistance change the total and bio-available sex steroids. There is a significant increase in bioactive IGF-I that inhibits systhesis of SHBG. Moreover, both insulin and IGF-1 can enhance the systhesis of androgens [46, 47]. Obesity also results in increased estrogen concentrations. Increase in androgens and estrogens, and decrease in SHBG and progesterone causes endometrial cancer [48].

## Conclusion and future work

The database has been designed to explore the molecular connections of obesity and its comorbid diseases. The data have been collected using text mining, and can be used to understand the complexity of developing comobid diseases in molecular level. Some pertinent questions in this regard are: How do the diseases develop with the onset of obesity, and is the obesity a primary disease condition to develop other diseses? The answers are still not clear. A complete understanding of obesity and its comorbid diseseas will enlighten the path towards drug development. Obesity causes hypoxia in adipose tissue and hypoxia induces alteration in gene expressions. Secretion of adipokines and adipose tissue specific hormones get altered. This affects end organs and causes a large number of diseases. Adipokines, transcription factors (TFs) and microRNAs (miRNAs) act as cross talk agents in the scenario of obesity. Exploring the adipokines, TFs and miRNAs will throw more light on molecular mechanism of comorbid disease development associated with obesity. Another important issue of developing obesity and its comorbid diseases is obesogens. Obesogens are foreign chemicals that are actively involved in the development of obesity epidemic. Obesogens may alter human metabolism using multiple mode of actions. The mechanism and effects of obesogens need to be explored to completely understand obesity - a lifestyle driven disease.

## Additional file

Additional file 1 Supplementary material. (PDF 66.9 kb)

## Abbreviations

AMPK: Adenosine monophosphate-activated protein kinase
ADIPOQ: Gene that coded for Adiponectin in human
APPL1: Adaptor protein, phosphotyrosine interacting with PH domain and leucine zipper 1
CASP: Caspases, or cysteine-aspartic proteases or cysteine-dependent aspartate-directed proteases
ER: Endoplasmic reticulum
FFA: Free fatty acids
FA: Fatty acids
FAT/CD36: FAT (fatty acid translocase), also known as CD36 (cluster of differentiation 36)
GO: Gene ontology
IRS: Insulin receptor substrate
IL, IL6: interleukin, interleukin 6
IFN- *γ*: Interferon- *γ*
IGF-1: Insulin-like growth factor-1
IGFBP: Insulin-like growth factor-binding protein
ICAM: Phosphatase and tensin homolog protein
IRE1: Phosphatase and tensin homolog protein
INS: Insulin
IL6: Interleukin-6
JNK: Phosphatase and tensin homolog protein
KEGG: Kyoto Encyclopedia of Genes and Genomes
LEP: Leptin
LPS: Lipopolysaccharide
MAPK3: Mitogen-activated protein kinase 3
NFKB1: Nuclear factor kappa B NAFLD: Non-alcoholic fatty liver disease
NFKB: Nuclear factor- *κβ*
OCDD: Obesity and co-morbid disease database
PPI: Protein-protein interaction
PLCG1: Phospholipase C, Gamma 1 gene
PTEN: Phosphatase and tensin homolog protein
STAT3: Signal transducer and activator of transcription 3- a transcription factor
SHBG: Sex hormone-binding globulin
T2D: Type 2 diabetes
TNF- *α*: Tumor necrosis factor- *α*
TLR: Toll-like receptor
TP53: Tumor protein
VEGFA: Vascular endothelial growth factor A
