# Supplementary material for: OCDD: an obesity and co-morbid disease database
Source: BioData Min. 2017 Nov 21;10:33. doi: 10.1186/s13040-017-0153-5 (PMC5697160; doi:10.1186/s13040-017-0153-5)
Supplement: Supplementary file 1 — Supplementary material. (PDF 66.9 kb) [file 13040_2017_153_MOESM1_ESM.pdf]

OCDD: An Obesity and Co-morbid Disease Database
Supplementary material

October 21, 2017

A brief note on Fisher exact test

Let us consider the following 2×2 contingency table as observed in an experiment.

	Number of Pubmed hits with disease but not obesity	Number of Pubmed hits with disease and obesity	Row Total
Disease A	a	b	$a+b$
Disease B	c	d	$c+d$
Column Total	$a+c$	$b+d$	$a+b+c+d(=n)$

The entries in the table are self-explanatory. Fishers exact test of independence on this data is performed in order to check whether the proportion of the Pubmed hits with disease A and obesity differs from that with disease B and obesity. In other words, the test indicates whether the degree of co-morbidity of obesity with disease A differs significantly from that with disease B. Now the probability of obtaining any such set of values in the table, keeping the marginals identical, is given by

$$P = \frac{(a+b)!(c+d)!(a+c)!(b+d)!}{a!b!c!d!n!} \tag{1}$$

This hypergeometric distribution is used to calculate the probability of getting the observed data, and all data sets with more extreme deviations, under the null hypothesis that the aforesaid proportions are the same. For the usual two-tailed test, the probability of getting deviations as extreme as the observed, but in the opposite direction, is also calculated. This is an exact calculation of the probability values. Unlike most statistical tests, there is no intermediate step of calculating a test statistic for which probability is approximately known.
